# Surgery of Bone

**Published:** 1904-11-19

**Authors:** 


### SURGERY OF BONE.

**The Ambulatory Treatment of Fractures of the Leg-bones.**—Openshaw[1] describes three cases—two in which the tibia and fibula were the seat of fracture, and one the femur. The method which has been in vogue on the Continent for ten years, consists in applying a strong leather splint which is moulded on a plaster cast. The limb is first enveloped from pelvis to ankle in plaster bandages, so as to make a casing a quarter of an inch thick. This is then cut off before it is quite set and surrounded by one more plaster bandage. In this case a plaster cast is formed, and on the cast the final splint is moulded in stout wet leather, steel bands being laid between the cast and the leather, which are afterwards riveted to the latter. The leather case laces up the front, and consists of a thigh-piece and knee-piece, leaving the knee bare. The thigh-piece, upon which the ischium rests, is fixed to two lateral metal supports, which serve to transmit the weight of the body to a steel sole-piece. The lateral supports may be jointed at knee and ankle. The patient is allowed to walk about directly this apparatus has been applied, and even in one of the worst cases of Pott's fracture which Openshaw had ever seen the ankle was quite mobile at the end of a month, when the bones were firmly united.

**Treatment of Fractured Patella.**—Martin and Thomas[2] divide cases of this injury into two classes. First, those in which there is not more than half an inch separation of the fragments, and in which there is but little tension in the joint; and second, those in which there is more than half an inch of separation when the knee is bent to a right angle or in which there is great fluid tension. For the first class, which includes most of the fractures resulting from direct injury, conservative treatment suffices. For the second, the open method of operation should be adopted at once. The tendinous expansion of the quadriceps is united by mattress sutures and the bone by silver wire.

**The Origin of Inflammatory Foci in Bone.**—Lexner[3] discusses the question as to why the incidence of tubercle and of acute osteo-myelitis falls upon different localities of bone. In experimentally produced disease, both tubercular and staphylococcal, infection falls upon the ends of the bone near the joint. This appears to depend on the arrangement of the vessels, which has been demonstrated by skiagrams of mercurial injections. There are three series of vessels in the marrow. The branches of the nutrient artery, which end near the epiphysis in a growing bone; small arteries entering the diaphysis near the epiphyseal line; and arteries entering through the epiphysis. Lexner believes that all foci in the epiphyseal regions are embolic. He considers that tubercular infection is produced by embolic clumps of bacilli, whereas pyogenic foci are not embolic, and this difference serves to explain the different locality of tubercular and pyogenic bone foci.

**Fracture of the Neck of the Femur.**—Marmaduke Sheild[4] insists on the great frequency with which this accident is overlooked. He advocates the practice of taking an immediate skiagram, and of not manipulating under an anæsthetic in any doubtful case, as imperative duties. The shortening, which is inconspicuous at first, becomes marked within a few weeks. In the aged the patient's life must be saved and the fracture left to take care of itself. In the middle-aged massage is the most important point in treatment and should be begun within the first week.

**Infantile Paralysis affecting the Shoulder-joint.**—Vulpius[5] relates experience gained in six cases of this affection in which he performed an operation to produce ankylosis of the joint. He argues that a substitution of the pectoralis and trapezius for the deltoid can do but little to remedy the paralysis. But if the humerus be fixed to the scapula, then good movements of the limb together with shoulder-blade can be obtained. He operates a year after the onset of the paralysis by a longitudinal anterior incision, removing all cartilage and synovial membrane and wiring the head of the humerus to the glenoid cavity.

**Myositis Ossificans Traumatica.** — Haga and Fugimura,[6] two Japanese surgeons, have observed several cases. In some the muscles of the arm were affected, in others the adductors, and in one the vastus internus. In all, the affection followed traumatism, and in those cases where portions were excised, microscopical examination showed blood extravasation, inflammatory reaction, fibrous tissue, cartilage, and true bone as successive stages in the transformation of the injured muscles. An experiment in which the thigh muscles of a guinea-pig were injured by repeated blows, showed that after several weeks ossification had taken place in the injured muscles. These facts all tend to prove that in the disease in question the ossification is primary in the muscle and does not arise as an outgrowth from the underlying bone.

**Osteitis Deformans.**—John B. Roberts[7] reports the case of a man aged 47. His father's father was said to have become bandy-legged in his old age; his father became very bent, and stooped. Fifteen years previously patient had broken the left humerus; it united readily, but he thought that present illness dated from that time. He complained of general feeling of lassitude and of a decrease in height amounting to $3\frac{1}{4}$ inches in ten years. There was a bulging backward of the whole lumbar region in place of the normal forward curve. The femurs were bowed, especially on the right side, the right clavicle and left humerus were uniformly enlarged and thickened. The left tibia had a thickened ridge extending from the tubercle. The back of the head appeared unnaturally large, and he said that he had had to increase the size of his hats. The writer suggests that the disease is essentially the same as leontiasis ossium, both being of the nature of tropho-neuroses.

[1] Lancet, March 12, 1904. [2] Med. Rec., June 11, 1904. [3] Edin. Med. Chir., 1904. [4] Clin. Jour., July 6, 1904. [5] Edin. Med. Chir., March 1904. [6] Ibid. [7] Phil. Acad. Sci. Trans., 1904, p. 437.

